# Le traitement chirurgical des fractures de la palette humérale chez l’adulte

**DOI:** 10.11604/pamj.2017.26.79.10781

**Published:** 2017-02-20

**Authors:** Redouane Hani, Mustapha Nekkaoui, Mohammed Kharmaz, Mohamed El Ouadghiri, Abdou Lahlou, Mly Omar Lamrani, Ahmed El Bardouni, Mustapha Mahfoud, Mohamed Saleh Berrada

**Affiliations:** 1 Service de Chirurgie Orthopédique, CHU Rabat, Ibn Sina Hospital, Maroc

**Keywords:** Fracture, palette humérale, ostéosynthèse, Fracture, humeral pallet, osteosynthesis

## Abstract

Le traitement des fractures de la palette humérale repose principalement sur la chirurgie de reconstruction par ostéosynthèse. On a colligé dans nos archives 40 cas de fracture de la palette humérale au service de traumatologie-orthopédie de CHU IBN SINA de RABAT du mois JANVIER 2012 au mois de DECEMBRE 2014, dont le but est de montrer les particularités cliniques, thérapeutiques et évolutives de ces fractures; ainsi que les difficultés de prise en charge de ces fractures complexes et d’évaluer les résultats. On a noté une nette prédominance masculine (75 % des cas) avec un âge moyen de 35 ans. Les étiologies sont dominées par accidents de la voie publique (56 %). Tous nos patients ont été hospitalisés par le biais des urgences puis opérés. Le type C selon la classification de MÜLLER et ALLGOWER(A.O) est le plus fréquent : 62,5% des cas. les lésions associées sont assez fréquents (52,5%) dans le cadre d’un poly-traumatisme. Tous nos patients ont été opérés (100% des cas) via un bord postérieur dans 70 % des cas. Ce geste comprenait une réduction puis ostéosynthèse par plaque de LECESTRE dans 82,5% des cas. Nos résultats sont de 85,5 % de bons et moyens résultats, conformément aux données de la littérature. La prise en charge de ce type de fracture est basée sur une réduction anatomique parfaite, par un montage solide qui doit permettre une rééducation précoce garante d’un bon résultat fonctionnel.

## Abstract (en)

The treatment of humeral pallet fractures is mainly based on reconstruction surgery with osteosynthesis. We collected the data of 40 patients with humeral pallet fracture from our archives in the Department of Orthopaedics and Traumatology at the Ibn Sina University Hospital, Rabat from january 2012 to december 2014. The aim of our study was to highlight the clinical, therapeutic and evolutionary features of these fractures as well as the challenges in managing these complex fractures and in evaluating the results. There was a clear male predominance (75% of cases) with an average age of 35 years. Etiologies were dominated by road accidents (56%). All our patients had an emergency hospital admissions and underwent surgery. Type C fracture, according to MÜLLER and ALLGOWER (A.O) classification was the most frequent: 62.5% of cases. Associated lesions were fairly frequent (52.5%) in polytraumas. All our patients underwent surgery (100% of cases), via posterior border in 70% of cases. This procedure included reduction and then osteosynthesis using a Lecestre-type plate in 82.5% of the cases. Our results were good and average in 85.5% of cases, according to literature data. The management of this type of fracture is based on perfect anatomic reduction, a solid assembly which should allow an early reeducation resulting in good functional result.

## Introduction

Les fractures de la palette humérale sont définies par les fractures situées entre l’insertion distale du muscle brachial antérieur et l’interligne articulaire du coude et sont classées dans les fractures de l’extrémité inférieure de l’humérus [1]. Les fractures de l’extrémité distale de l’humérus (FEDH) représentent 1 à 2% des fractures de l’adulte selon Morrey [2], ils peuvent être extra- ou intra-articulaires. Dans ce dernier cas, elles peuvent être complexes du fait de la comminution et/ou de l’état porotique de l’os. Leur habituelle complexité anatomique a longtemps conditionné la diversité de leurs traitements, et leur prise en charge demeure encore très difficile [3]. Si le traitement orthopédique reste parfois de mise pour les fractures non déplacées, ou exceptionnellement, pour les grands fracas, la chirurgie est considérée aujourd’hui comme le traitement privilégié. Elle doit préférer l’ostéosynthèse la plus stable possible, afin d’éviter tout démontage et permettre une mobilisation précoce, seul moyen d’éviter une raideur du coude, complication la plus fréquente.

## Méthodes

Nous rapportons une étude prospective de 40 cas de fractures de l’extrémité distale de l’humérus traitées chirurgicalement et suivies au Service de chirurgie orthopédique et traumatologie CHU IBN SINA RABAT Maroc sur une période de 3 ans du janvier 2013 jusqu’au décembre 2015. Les patients étaient inclus dans l’étude selon les critères suivants: la survenue d’une fracture de l’extrémité distale de l’humérus, non pathologique, chez des patients de plus de 16 ans. L’objectif de l’étude est d’évaluer et analyser les résultats fonctionnels ainsi que les complications après un traitement chirurgical conservateur chez les patients de plus de 16 ans.

## Résultats

L’âge de nos patients variait entre 17 et 77ans. La moyenne d’âge globale de survenue de la fracture humérale est de 38 ans. La moyenne d’âge des femmes est de 45 ans, celles des hommes et de 35 ans. On note que le taux de fractures est plus élevé chez les patients entre 17ans et 30 ans (40%), avec 32% entre 30-50ans ans et 20 % entre 50-70 ans et 8% entre 70-77ans. Les atteintes les plus fréquentes se voient chez des sujets de la deuxième à la troisième décade, ceci veut dire que c’est la population jeune qui est la plus exposée. L’étude comprend 30 hommes (75%) 10 femmes (25%) pour soit un sex-ratio homme- femme de 1/3 la prédominance masculine est significative. Dans notre série le côté dominant été le côté droit chez 30 patients soit un pourcentage de 75%. Dans notre série, prédomine l’atteinte du côté droit dans 22 cas soit un pourcentage de 55% par rapport à 17 cas au côté gauche soit un pourcentage de 42%. Seul un Sujet ayant présenté une atteinte bilatérale. La cause la plus fréquente des fractures de la palette humérale est représentée par les accidents de la voie publique avec un pourcentage de 56% de l’ensemble de 40 cas, suivis par les agressions avec un pourcentage de 20% et les chutes avec pourcentage de 13%, les accidents du sport représentent 11%. Dans notre série, on note une prédominance de l’impact direct dans la genèse des fractures de la palette humérale (65%), alors que le mécanisme indirect est retrouvé dans 14 cas soit 35% des cas, ceci est expliqué par la fréquence des accidents de la voie publique et les agressions. Tous nos patients se sont présentes aux urgences avec une attitude du traumatisé du membre supérieur avec coude en semi-flexion a 90°. L’ouverture cutanée a été classée selon la classification de CAUCHOIX et DUPARC. Elle a été notée chez 15 patients (37.5%). Aucune lésion vasculaire n´a été rapportée, Nous avons relevé 2 cas de paralysie du nerf radial.

**Figure 1:**
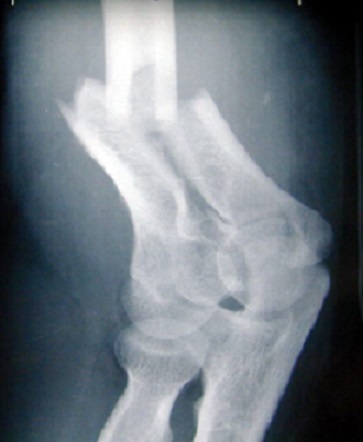
Fracture sus et intercondylienne de la palette humérale

**Figure 2:**
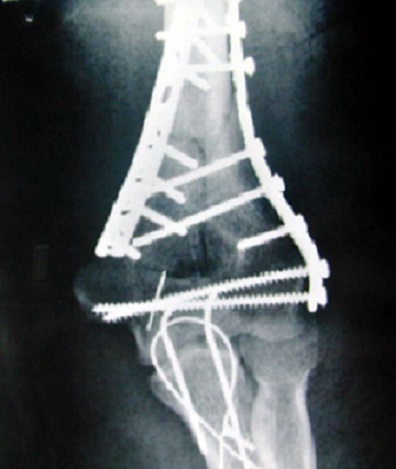
Ostéosynthèse par plaque externe type Lecestre et plaque 1/3 tube associée à un vissage condylaire direct+embrochage haubanage de l’olécrane

Dans notre étude pour classer les 40 cas de fractures de la palette humérale on a adopté la classification de l’AO. Elle distingue les fractures articulaires et extra articulaires pures en 3 catégories (A,B,C). A : fracture extra-articulaire, B : fracture articulaire partielle, C : fracture articulaire totale (Figure 1). Ainsi on peut constater la prédominance des fractures sus et inter condyliennes qui représentent 63% des cas de notre série, suivies des fractures supra condyliennes 25%; alors que les fractures parcellaires articulaires sont plus rares, observés seulement chez 12% des patients. Tous nos patients ont été traités chirurgicalement par la voie d’abord postérieure avec une ostéotomie de l’olécrane. La fixation par plaque de LECESTRE seule ou associée à d’autres matériels (une plaque 1/3 tube et ou vissage et ou embrochage) était la méthode la plus utilisée avec un pourcentage de 82,5% de l’ensemble des matériaux utilisé, suivie par l’embrochage seul dans 7,5% des cas (Figure 2).

**Figure 3:**
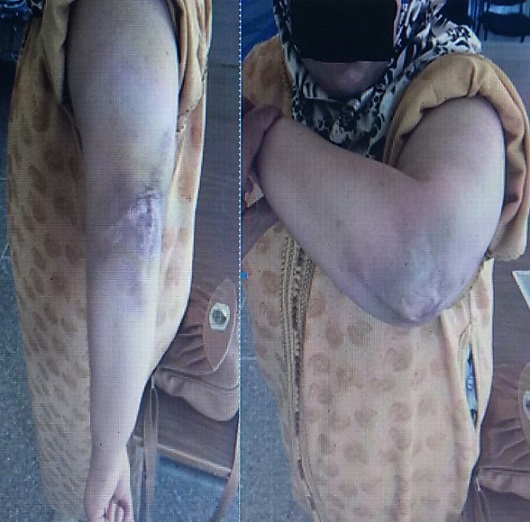
Les résultats fonctionnels de cette patiente à 2 ans sont satisfaisants avec une bonne récupération des amplitudes du coude, sans douleur

Le drainage et l’antibio-prophylaxie étaient systématiques chez tous les patients, en plus d’une immobilisation par une attelle plâtrée brachio-antébrachiale maintenue en moyenne 3 semaines jusqu’à l’atténuation des phénomènes douloureux et inflammatoires. Un cas de fistulisation du site opératoire (fistule ramenant des sérosités au niveau de la face postérieur du coude) a été observé un mois après l’intervention chez un patient âgé de 41ans, diabétique, ayant régressé après l’administration d’un traitement antibiotique adapté. Un autre cas d’infection du site opératoire chez un patient âgé de 37ans sans antécédents particuliers. 2cas de paresthésie du nerf ulnaire, qui peuvent être expliqué par la neurolyse extensive. Dans notre série on a trouvé 09 cas de raideurs soit 22,5 % des cas dont 4 cas ont bénéficié d’une arthrolyse chirurgicale, sachant que Le coude est une articulation qui supporte mal l’immobilisation. Les causes qui peuvent être à l’origine de cette raideur : une immobilisation prolongée, la complexité de la fracture, ou l’absence de rééducation. On note dans notre série 07cas de cal vicieux soit 17,5% des cas, ceci est dû à un défaut de la réduction des fractures complexes, ainsi que la difficulté de retrouver les repères osseux. On note la présence de 2 cas de pseudarthrose soit 5%, (1 cas de pseudarthrose septique). L’arthrose post traumatique est la rançon tardive de toute fracture articulaire imparfaitement réduite, dans notre série on a constaté 8 cas d’arthrose du coude (Figure 3).

## Discussion

La plupart des auteurs rapportent dans leurs séries la nette prédominance de la lésion chez la population jeune et de sexe masculin. L’étude du registre national Ecossais nous apporte des données épidémiologiques très intéressantes [4] par l’importance de la population étudiée (595 000 personnes). L’incidence globale retrouvée des fractures de l’humérus distal est de 5.7/100000/an avec une distribution bimodale : le premier pic d’incidence se situe chez l’homme de 12 à 19 ans, le deuxième pic se retrouve chez la femme de plus de 80 ans (Figure 2). Il est trouvé un âge moyen de fracture de 36.8 ans (12-87) chez l’homme et de 59.7 ans (13-99) chez la femme. On note une discordance entre les résultats des différents auteurs concernant la prédominance du côté droit ou gauche. Pour les circonstances du traumatisme, on note la prédominance des accidents de la voie publique dans notre étude, alors que l’étiologie principale dans les autres séries est représentée par la chute. On constate que dans toutes les séries étudiées les fractures sus et inter-condyliennes (type C) sont les plus fréquentes des fractures de la palette humérale, suivies par les fractures supra-condylienne (type A). Aussi, nos résultats sont en accord avec ceux décrits par les différentes séries. L´aspect clinique se résume à un gros coude douloureux masquant la palpation des repères normaux classiques que sont la ligne de Hunter (ligne joignant l´olécrâne aux épicondyles, coude en extension) et le triangle isocèle de Nélaton (surface cutanée déterminée par la position respective de l´olécrâne et des épicondyles, coude en flexion [1]. Le diagnostic repose sur l´analyse des clichés du coude de face en extension et de profil à 90° de flexion. Ces clichés sont cependant souvent de mauvaise qualité en raison du caractère très algique de la fracture ne permettant pas la réalisation d´incidences orthogonales correctes, chez un patient immobilisé dans une attelle. Ils seront souvent refaits au bloc opératoire [4]. Le scanner devient de plus en plus systématique, notamment pour les fractures articulaires. Chez l´adulte, l´échographie n´a pas sa place dans le diagnostic positif. En revanche, à distance, en association avec l´IRM, elle participe au bilan lésionnel ligamentaire [1]. La complexité anatomique de l’extrémité inférieure de l’humérus, la comminution souvent fréquente de ces fractures, ainsi que la proximité du nerf radial et cubital associées à la multiplicité des formes anatomopathologiques sont autant de raisons qui font que ces fractures posent un réel problème thérapeutique pour le chirurgien traumatologue. Si le traitement orthopédique reste parfois de mise pour les fractures non déplacées, ou exceptionnellement, pour les grands fracas, la chirurgie est considérée aujourd’hui comme le traitement privilégié. Elle doit préférer l’ostéosynthèse la plus stable possible, afin d’éviter tout démontage et permettre une mobilisation précoce, seul moyen d’éviter une raideur du coude, complication la plus fréquente. Les ostéosynthèses par broches ou vis isolées ou association des deux ont été progressivement abandonnées du fait de leur précarité et, depuis le consensus de la table ronde de la SOFCOT de 1979 [5], les montages par plaques vissées sont reconnus comme étant le traitement de choix. Cependant, le type et la localisation optimale des plaques restent un sujet de controverse [5].

Depuis 1992 une plaque en Y a été mise en place « jambes vers le bas » appelée « plaque Lambda ». Les qualités biomécaniques de cette plaque ont été démontrées par Fornasiéri et al. en 1997 [6], si bien que depuis 1992 nous l’utilisons chaque fois que nécessaire dans les fractures de l’extrémité distale de l’humérus quelles soient articulaires, extra-articulaires ou diaphyso-métaphysaires [3]. Le taux de déplacement fracturaire avec démontage était de 14% dans notre série, il a intéressé cinq patients. Le symposium de la SOFCOT en 2007 souligne un taux comparable de 16 %. Les études les plus récentes, respectant les principes, à présent connus, d’une ostéosynthèse stable, montrent des taux inférieurs variant entre 0 % et 10 % [7]. Notre série présente un taux d’infection de 5% qui est comparable à celui de la majorité des séries étudiées, 5 % chez Raiss [8] et 8 % chez Lahdidi [9]. Ces infections peuvent être dues selon Jupiter au délai d’intervention ainsi que sa durée et les modalités d’ostéosynthèse (plus grande fréquence d’infection après ostéosynthèse par plaque) [10]. La pseudarthrose a été observée dans 5% des cas, tout comme en témoigne l’ensemble des séries nationales et étrangères, 5,1% pour Nedellec et 3,6% pour Raiss. Un montage peu rigide en est bien souvent la cause principale. Le nerf le plus fréquemment atteint est le nerf ulnaire, ce qui concorde avec nos résultats : 2 cas de paresthésie du nerf ulnaire. Raiss [8] a décrit 8 cas de lésions nerveuses du nerf ulnaire, alors qu’OBERT [11] en a relevé 6 cas. L’atteinte du nerf ulnaire est due à son anatomie qui le rend particulièrement vulnérable dans cette région. La raideur a été trouvée dans 23,63% des cas chez Raiss, 16% chez Roques, 20% chez Obert, 8% chez Lahdidi, la fréquence des raideurs dans la majorité des séries reste notable, notre série a noté 09cas de raideurs avec une fréquence de 22,5%, cette fréquence qui reste assez importante est en rapport avec non suivie de la rééducation par les patients. Nos résultats étaient satisfaisants dans 85%, un taux qui rejoint les résultats trouvés par Jupiter (77%) et Saragaglia (68%), ce qui confirme en accord avec la littérature l’intérêt d’une prise en charge chirurgicale des fractures de la palette humérale dont le pronostic fonctionnel repose sur la restitution anatomique parfaite et les possibilités de mobilisation précoce.

## Conclusion

Les fractures de la palette humérale sont de plus en plus fréquentes, ceci est en rapport avec l’augmentation des accidents de la voie publique et leur violence. Le traitement chirurgical, par un abord postérieur de préférence, reste difficile compte tenu de la comminution souvent rencontrée, c’est pour cette raison que seule une ostéosynthèse solide et stable permet la restitution anatomique du coude, en adaptant les indications aux types de fractures et en utilisant une technique plus codifiée, pour permettre une rééducation précoce, meilleur garant de la récupération de la fonction du coude.

### Etat des connaissances actuelles sur le sujet

- Fractures fréquentes du sujet jeune;
- Prise en charge souvent difficile;
- Complications assez fréquentes.

### Contribution de notre étude à la connaissance

- La plaque Lecestre est l 'option de choix de l'ostéosynthèse des fractures sus et intercondyliennes;
- La raideur reste la complication redoutable des fractures articulaires de la palette humérale;
- Le pronostic fonctionnel repose sur la restitution anatomique parfaite et la mobilisation précoce.
